# Three Decades of Enamel Matrix Derivative: From Dental Innovation to Extra‐Oral Applications

**DOI:** 10.1155/ijod/6556335

**Published:** 2026-06-02

**Authors:** Faustino Mercado, Carolina Loch, Ahmad Mustafa, Euna Lee, Michelle Hecker

**Affiliations:** ^1^ Faculty of Dentistry, University of Otago, Dunedin, New Zealand, otago.ac.nz; ^2^ Sir John Walsh Research Institute, University of Otago, Dunedin, New Zealand, otago.ac.nz

## Abstract

**Introduction:**

Enamel matrix derivative (EMD; Emdogain) has been deployed for three decades as a biologically active material in periodontal regeneration. Widely adopted in periodontology, its indications are expanding into endodontics, ridge preservation, implantology, dermatology and oncology‐related indication. Contemporary applications encompass vital pulp therapy, alveolar ridge preservation (ARP), peri‐implantitis adjunctive therapy and soft‐tissue wound healing.

**Materials and Methods:**

This narrative review synthesises English‐language literature (1996–2025) retrieved from PubMed, Scopus and Cochrane Oral Health. The scope prioritised clinical outcomes in endodontic and periodontal contexts—recession coverage, intrabony and furcation defects, osteogenesis, peri‐implantitis management and wound healing. Included were clinical trials (preferentially randomised controlled trials [RCTs]) with ≥6 months follow‐up; selective animal studies were incorporated to clarify tissue‐level and histologic mechanisms, particularly for endodontic applications.

**Results:**

Accumulated evidence indicates EMD augments the probability of complete root coverage (RC) and increases keratinised tissue (KT) width in mucogingival procedures. EMD, alone or adjunctive to bone grafts or membranes, improves clinical parameters in intrabony and certain furcation defects and demonstrates osteopromotive effects in preclinical and clinical models. Adjunctive use in peri‐implantitis and implant‐site regeneration shows promising amelioration of inflammatory and bone‐regenerative endpoints. Conversely, EMD has limited efficacy for preservation of alveolar ridge dimensions post‐extraction. In vital pulp therapy, EMD supports tertiary dentinogenesis and dentine‐bridge formation. Mechanistically, EMD enhances cell migration, proliferation, extracellular matrix (ECM) deposition and angiogenesis. Preliminary data indicate potential utility in non‐healing cutaneous ulcers, though evidence remains sparse.

**Conclusion:**

EMD is a validated adjunct in periodontal regeneration—particularly recession coverage and the management of intrabony defects—with encouraging osteogenic and reparative properties that extend to endodontic and wound‐healing indications. Future investigations should standardise dosing/regimens, explore controlled‐release and combination modalities and evaluate long‐term clinical effectiveness and cost‐benefit metrics.

## 1. Introduction

Enamel matrix derivative (EMD) was introduced in 1996 and later approved by the United States Food and Drug Administration for treating periodontal defects and gingival recessions. It is initially marketed as Emdogain by Biora and acquired by Straumann in 2003, EMD has been extensively studied for its role in periodontal tissue regeneration. Although outcomes vary, EMD is among the few biomaterials shown to achieve true periodontal regeneration by stimulating the formation of cementum, periodontal ligament (PDL) and alveolar bone [1, 2]. EMD consists of approximately 90% amelogenins (AMELs). Consequently, its biological activity is primarily attributed to this enamel protein [3]. EMD’s continued use is largely due to its status as the only chairside biologically active product for several years. Recently, new biologically active materials have emerged, offering potential improvements in regeneration, wound healing and clinical outcomes. Whether EMD will remain a material of choice or take on a more specialised role remains to be seen. EMD’s versatility has led to research into broader applications, including wound healing, bone grafting, dental implantology, endodontics, pulpal therapy and dental traumatology. Studies are also exploring its effects on cancer cells and its potential impact on tumour growth and metastasis [4–6]. This review critically evaluates the evolving role of EMD across various dental and medical applications, highlighting both its established benefits and emerging therapeutic possibilities.

## 2. Methods

A narrative literature search was conducted with the goal of providing a summary or critical analysis of the topic. Here, the narrative literature review aimed to identify clinical and preclinical evidence on EMD use in endodontic, periodontal, implant and dermal wound healing applications. Our search strategy included electronic databases (Medline, PubMed, Scopus and Google Scholar), which were searched for studies published between 1996 and 2025, supplemented by Cochrane Oral Health Group/Cochrane Library searches (1996–2025). Search keywords were predefined and applied individually and in combination, and included the following terms: ‘enamel matrix derivative’, ‘EMD’, ‘Emdogain’, ‘periodontal regeneration’, ‘intrabony defect’, ‘furcation’, ‘randomised clinical trial’, as well as endodontic terms such as ‘pulp capping’, ‘root resorption’, ‘regeneration’, and ‘revascularisation’, in addition to ‘peri‐implantitis’ and ‘wound healing’.

Inclusion criteria comprised clinical studies evaluating EMD, with emphasis on randomised controlled trials (RCTs), a minimum follow‐up of 6 months, and publication in English. Exclusion criteria included non‐clinical studies (except where animal studies provided essential histological evidence), studies without relevant clinical outcomes, duplicate publications and reports lacking sufficient methodological detail. Animal studies were included selectively where tissue‐level outcomes were required to support clinical interpretation, particularly in endodontic applications. The study selection process involved a two‐stage screening methodology. First, titles and abstracts were screened for relevance to EMD. Second, full‐text articles were assessed against predefined inclusion and exclusion criteria. Reference lists of key systematic reviews, major trials, and Cochrane reviews were also screened to identify additional eligible studies. Data extraction was performed using a structured approach, capturing study design, population or defect type, interventions and comparators, follow‐up duration and primary outcomes, including probing depth (PD) reduction, clinical attachment level (CAL) gain, radiographic findings, adverse events and, where applicable, histological endpoints. Findings were synthesised narratively, with greater emphasis on well‐designed RCTs, studies with longer follow‐up, and evidence included in Cochrane‐level reviews.

### 2.1. The Origin and Evolution of AMEL

The duplication and diversification of ancestral genes encoding the formation of mineralised tissues—bone, dentine and enamel—were key events in the evolution of vertebrates, allowing for protection and locomotion (bones) and for feeding adaptations (teeth). Mineralised tissues emerged early in the history of vertebrates, as they were already present in jawless fish 500 million years ago [7–9]. Teeth probably originated from odontodes, which composed the dermal armour in these early vertebrates, when jaws first formed in early gnathostomes— jawed vertebrates [9]. Teeth are present in most vertebrate lineages, sharing a typical structure comprising a highly mineralised protective tissue (enamel or enameloid) covering a living tissue (dentine) which surrounds a pulp cavity containing nerves and blood vessels [9]. In mammals, enamel is deposited by epithelium‐derived cells (ameloblasts) and is composed of three main enamel matrix proteins (EMPs): AMEL, accounting for 90% of the enamel matrix, ameloblastin (AMBN), and enamelin [10]. AMEL is a non‐glycosylated extracellular matrix (ECM) protein that initiates and regulates the growth of hydroxyapatite crystals during development. It plays an important role in the structure and stability of enamel, as exemplified by severe structural defects on the enamel of mouse knockout models and patients with X‐linked amelogenesis imperfecta [8, 11]. Genetic studies have proposed that the origin of the AMEL gene was derived from AMBN duplication, and this happened long before the evolution of tetrapods–four‐legged vertebrates [9]. Enamel covers the teeth of lobe‐finned bony fishes and tetrapods and covered the scales and dermal bones of fossil lobe‐finned fish. Other ray‐finned fish have enamel homologues covering their scales and dermal bones. Fossil and genetic evidence suggest that enamel originated on the dermal skeleton, probably on the scales of bony fishes [12]. This suggests that EMPs are widely present and conserved across species and are not only confined to the oral cavity but also present in dermal structures covering the body [13]. The presence of AMEL in a variety of vertebrates, in the dentition and elsewhere, could potentially explain why EMD products have applications in diverse areas such as periodontal regeneration, keratinised tissue (KT) improvement, wound healing and bone formation.

### 2.2. The Role of AMEL in Tooth Development

AMEL makes up 90% of EMP and plays a major role in enamel mineralisation and morphological changes. AMEL is comprised of two self‐assembly regions: a hydrophobic amino‐terminal domain A and a hydrophobic carboxy‐terminal domain B. AMEL binds to hydroxyapatite crystals by the hydrophilic domain B [14]. AMEL molecules commonly form nanospheres due to the binding capacity of both A and B terminals. The central region of the AMEL molecule (C‐domain) forms a dense central area of nanospheres surrounded by the long tails of both terminals [14, 15]. The formation of these AMEL nanospheres seems to be associated with the spacing of enamel crystallites, and the size of these nanospheres and their close interaction with the crystal surface are implicated in the width and thickness of enamel crystals. After secretion, AMELs are rapidly processed in a stepwise and controlled manner and are removed during late maturation. The degradation of enamel matrix components (mostly AMELs) is concurrent with the growth of enamel crystals, creating a highly organised enamel layer that is predominantly inorganic [14].

### 2.3. The Role of the Hertwig’s Epithelial Root Sheath (HERS) in Root Formation

After crown formation in tooth development, the inner and outer enamel epithelia fuse below the cervical margin to form HERS [16, 17]. HERS is essential for root formation, as it subdivides dental ectomesenchymal tissues into the dental papilla and dental follicle, regulates the timing of root development, induces mesenchymal cell differentiation into odontoblasts and cementoblasts and serves as a precursor for cementoblasts [17]. HERS also regulates and maintains the space and function of the PDL [16]. Past studies have supported the role of HERS in cementum formation and the notion that HERS‐derived products are implicated in the formation of enamel‐related proteins, which might initiate the production of acellular cementum [18, 19]. Other studies have also supported the role of EMPs in cementogenesis and that the HERS is involved in the development of both acellular and cellular cementum [18, 20]. Hammarstrom suggested that a makeshift porcine enamel matrix could initiate the formation of a tissue‐like acellular extrinsic fibre cementum in monkeys and that exogenous enamel matrix had induced the same type of tissue reaction as an endogenous enamel matrix on rat molars [1]. This has resulted in the development of a clinical product used for periodontal regeneration, KT enhancement, wound healing and bone formation. EMD contains high levels of EMPs, particularly AMEL, based on their proposed role in cementogenesis. However, the role of EMPs in cementum and attachment apparatus formation remains unclear. Some studies question the presence of enamel proteins in cementum or the transcription of AMEL in cementum [17, 21–23]. Other research suggests that enamel‐related proteins such as AMBN and enamelin may also be involved [17, 24]. Studies using developing porcine teeth found immunocytochemistry labelling for AMEL, but not AMBN, in developing roots; however, these findings do not establish a causal relationship between EMD and cementogenesis [25]. AMELs are highly conserved proteins that have evolved over thousands of years [26]. As a result, allergic or immunogenic reactions have not been reported in clinical studies involving EMD [27–30]. In a Swedish study of 247 patients treated surgically with EMD, serum samples were analysed for total and specific antibody levels [30]. No patients, including those with allergies, showed immunologic reactions to EMD in haematological examinations after its application, confirming the product’s safety [30].

### 2.4. EMD

**Figure 1:**
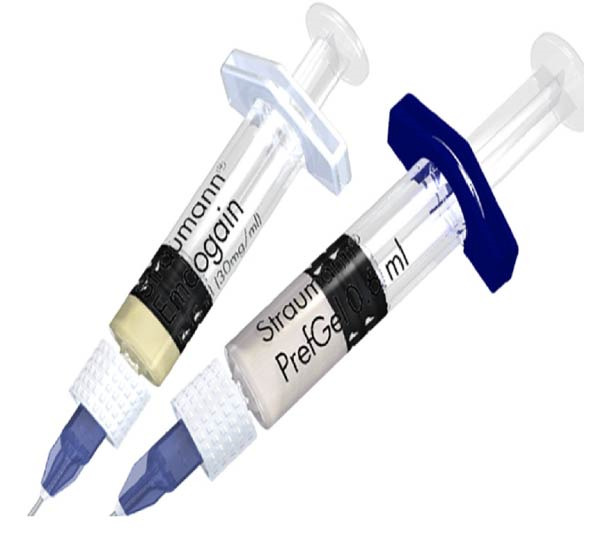
EMD and Prefgel. EMPs mainly amelogenin commercially available as Emdogain, sold with surface conditioner, Prefgel, which is 24% EDTA.

EMD describes the purified extracts of EMPs from developing teeth of minipigs. These EMPs are hydrophobic and insoluble in nature, maintaining their crystal form in vivo and in vitro. The commercially available product EMD contains purified powderised EMPs extracted from porcine tooth buds dissolved in a vehicle propylene glycol alginate, packaged in a syringe (Figure 1). EMD consists primarily of AMEL (90%) and a group of non‐AMELs, including AMBN, enamelin, tuftelin and proteolytic enzymes [31]. EMD also comes with Prefgel, a 24% ethylenediaminetetraacetic acid (EDTA) solution, which is used to ‘condition’ the root surface by removing the smear layer prior to the application of EMD.

### 2.5. Clinical Applications of EMD

#### 2.5.1. Endodontic Applications of EMD

Within endodontics, EMD has attracted interest due to the biological activity of AMELs, which are thought to regulate odontoblast differentiation and dentine formation [32]. This biological rationale has led to the promotion of EMD as a pulp capping material in vital pulp therapy procedures, including pulpotomy and direct pulp capping (DPC), particularly in cases of pulpitis or iatrogenic pulp exposure during restorative interventions [33–35]. The overarching aim of these procedures is to preserve pulp vitality by facilitating healing of the dentine–pulp complex. This is achieved through the removal of inflamed tissue, followed by the application of a bioactive material capable of stimulating mesenchymal stem cells within the pulp to differentiate into odontoblast‐like cells. These cells subsequently deposit tertiary dentine, forming a mineralised barrier that protects the underlying pulp from further insult. Experimental and clinical studies in both animal and human models have demonstrated that EMD, when used as a DPC material, can induce the formation of dentine‐like hard tissue [36–38]. In some reports, this response exceeded that observed with traditional pulp capping materials such as calcium hydroxide [36–38]. However, the outcomes are not uniformly favourable. While several investigations have documented successful dentine bridge formation [36–39], others have reported undesirable effects, including diffuse or ectopic hard tissue deposition along pulpal walls, leading to narrowing of the pulp chamber, or the presence of irregular calcifications within otherwise healthy pulp tissue [39–43].

Indeed, several studies have highlighted inconsistent histological outcomes, with only a proportion of treated teeth demonstrating organised dentine bridge formation, while others exhibited disorganised or incomplete calcification patterns [44–48]. Comparative studies further suggest that relative to other contemporary pulp capping materials such as mineral trioxide aggregate (MTA), biodentine and DYCAL, EMD may result in fewer odontoblast‐like cells and lower rates of dentine bridge formation [45].

The inflammatory response elicited by EMD in pulp tissue also appears variable. Some studies have reported healing responses consistent with physiological wound repair [37–39, 44, 48], whereas others have observed persistent moderate to severe inflammation that may compromise pulp healing and negatively influence prognosis [42, 49]. Animal studies, particularly in canine models, have demonstrated that such inflammation can disrupt the odontoblastic layer and damage both dentine and surrounding pulp tissue [41, 50]. These pathological changes may lead to complications including discontinuity of the lamina dura, periapical bone resorption, external root resorption and failure of dentine bridge formation [51].

The heterogeneity of these findings is likely attributable to substantial methodological variation across studies, including differences in animal models, sample sizes, extent of pulp removal, restorative protocols and duration of follow‐up, which has ranged from 28 days to 24 months. Such variability limits direct comparison and complicates interpretation of EMD’s true histological and clinical performance in DPC.

Clinical investigations in both permanent and primary human dentitions have similarly yielded mixed results, with most studies focusing on clinical and radiographic outcomes rather than detailed histological assessment. In permanent teeth scheduled for orthodontic extraction, EMD has been associated with fewer post‐operative symptoms compared with calcium hydroxide [39, 42]. Similarly, in permanent molars with reversible pulpitis, EMD has demonstrated reduced post‐operative pain and decreased reliance on anti‐inflammatory medications when compared with MTA, biodentine and calcium hydroxide [52].

In primary teeth affected by caries, DPC using EMD has shown clinical success rates ranging from 83% to 100% and radiographic success rates between 60% and 100% [53–56]. Comparative studies indicate that EMD may offer improved radiographic outcomes relative to formocresol, as reported by Sabbarini et al. [54], although other investigations have found no statistically significant differences between EMD and conventional pulp capping materials [53, 55, 56] (Table 1).

**Table 1:** EMD and vital pulp therapy (in‐vivo, DPC).

Publication	Sample	Procedure	Type of restoration	Follow‐up period	Result	Conclusion
Al‐Hezaimi et al. [57]	Baboon 32 premolars (8 teeth each)	Direct pulp capping	RMGIC	4 months	EMD‐CaOH = 2.02 ± 0.15 mm EMD‐MTA = 2.05 ± 0.31 mm EMD‐PC = 1.91 ± 0.28 mm	MTA produces a better quality reparative hard tissue response with the adjunctive use of EMD
Darwish et al. [50]	Dog 30 (15 per group) immature permanent teeth	Pulpotomy	GIC + amalgam	1, 2 and 3 months	0.4 to 0 mm	Observed root apex closure by cementum formation and normal PDL attachment to new alveolar bone. EMD not effective for dentino–pulpal complex regeneration due to inflammation and pulp degeneration
Sabbarini et al. [48]	Human 10 carious primary canines	Pulpotomy	GIC	1 and 2 weeks and 6 months	Mean thickness measured in 3 central sections for the teeth: 0.137 ± 0.105 mm; 0.021 ± 0.004 mm; 0.276 ± 0.905 mm	Process resembling classic wound healing with subsequent neogenesis of normal pulp tissue
Kaida et al. [36]	Rat 90 first molars (30 w/EMD)	Pulpotomy	GIC	7, 14 and 28 days	~ 0.3 mm^2^	More reparative dentine formed with EMD than Vitapex or control
Nakamura et al. [37]	Pig (adult) 22 (11 w/EMD) premolars	Direct pulp capping	GIC	2 and 4 weeks	7–8 mm^2^	Twice as much dentine‐like hard tissue formed in EMD‐treated teeth compared to CaOH‐treated teeth
Nakamura et al. [38]	Pig (adult miniature) 36 teeth (12 in EMD)	Pulpotomy	GIC	Observed over: 3, 4 and 8 weeks	1.292 ± 0.349 mm 1.513 ± 0.731 mm^2^	No adverse effects and significantly thicker dentine‐like bridge in EMD‐treated teeth than CaOH
Nakamura et al. [58]	Pig (adult miniature) 72 permanent lower incisor teeth	Pulpotomy	GIC	Observed over: 4 days and 1, 2, 3, 4, 8 and 12 weeks	EMD 1.928 ± 0.434532 mm 2.419 ± 0.8438 mm^2^ CaOH 0.859 ± 0.2911 mm 1.628 ± 0.4956 mm^2^	EMD produced significantly more reparative dentine than CaOH and showed faster initiation of healing
Olsson et al. [39]	Human 9 pairs (18) of contralateral premolars scheduled for orthodontic extraction	Pulpotomy	Teflon disc + ZOE cement + GIC	12 weeks	472,000 µm^2^	New pulp tissue filled the space initially occupied by the gel. Hard tissue formed along dentine walls and in patches in pulp
Orhan et al. [47]	Rats 144 (24 w/EMD) incisors	Direct pulp capping	IRM	7 and 28 days	99–199 µm	Reparative dentine formed in less than 60% of cases; along the root canal walls Smallest number of odontoblast‐like cells in EMD group than CaOH, MTA, PRP
Bajić et al. [44]	Vietnamese pig 20 (10 each)	Direct pulp capping	GIC	28 days	–	Results ranging from presence of incomplete dentine bridge to complete closure of pulp chamber, inflammation of pulp mild to moderate, angiogenesis and observed. No necrosis or bacteria present in pulp
Bollu et al. [49]	Human 60 (20 per group) premolars scheduled to be extracted for ortho treatment	Direct pulp capping	RMGIC	15^th^ or 45^th^ day	–	MTA and MTA/EMD combination produced better quality hard tissue response compared with EMD. More chronic inflammatory infiltrate in EMD treated teeth with calcified material present above the pulpal floor
Devi et al. [45]	Human 78 (26 EMD) permanent teeth indicated for surgical extraction	Direct pulp capping	IRM + RMGIC + GIC	6 weeks	–	No calcific bridge formation. Higher proportion of teeth treated with EMD with severe inflammatory response
Fransson et al. [40]	Human 18 (9 w/EMD) premolars scheduled for extraction for orthodontic treatment	Pulpotomy	Teflon disc + ZOE cement + GIC	12 weeks	–	Hard tissue formed along dentine wall, no dentine bridge formation
Igarashi et al. [46]	Rat 47 first molars (unknown number treated with EMD)	Pulpotomy	GIC	Day 4, 7, 14, 30	–	Dentine bridge formation in 27.3%. In incomplete bridge, osteodentine and diffuse calcification throughout pulp chamber
Ishizaki et al. [41]	Dog 32 (24 w/EMD) canines	Direct pulp capping	Zinc‐phosphate cement + amalgam	1, 4 and 8 weeks	–	Dentine formation along root canal wall, disappearance of odontoblasts, atrophy of pulp cells and vascular degeneration
Kiatwateeratana et al. [42]	Human 26 (13 w/EMD) premolars scheduled for orthodontic extraction	Pulpotomy	IRM + RMGIC + GIC	1 day, 2 weeks, 3 and 6 months	–	No complete dentine bridge formation in EMD‐treated teeth. CaOH more favourable
Silva et al. [51]	Dog 40 (24 w/EMD) teeth	Pulpotomy	Amalgam	7 and 70 days	–	Radiographic PDL widening, mild to moderate inflammation, followed by necrosis of remaining pulp and apical periodontitis. No dentine bridge formation
Garrocho‐Rangel et al. [53]	Human 90 (45 w/EMD) decayed primary molars	Direct pulp capping	Dentine adhesive + Vitrebond glass ionomer + metallic crown	1, 6 and 12 months	97.8% clinical and radiographic success	No significant differences to CaOH
Sabbarini et al. [54]	Human (aged 4–7) 30 (15 pairs) carious primary molars (15 treated with EMD)	Pulpotomy	GIC + SSC	2, 4 and 6 months	93% clinical and 60% radiographic success	EMD is a promising material that may be as good or better than Formocresol
Singh et al. [52]	Human 93 (24 w/EMD)	Pulpotomy	GIC, replaced by resin composite after 1 week	Symptoms: 6, 12 and 24 h Clinical and radiographic: 1, 3, 6 and 12 months	100% clinical and radiographic success	No statistically significant difference in clinical and radiographic success between EMD, CaOH, MTA, Biodentine. Statistically significant lower post‐op pain and use of anti‐inflammatory medication
Sunitha et al. [55]	Human 72 (18 w/EMD)	Pulpotomy	SSC	6, 12, 18 and 24 months	Clinical 83% and 72% radiographic success	No statistically difference in clinical and radiographic success between Formocresol, Pulpotec, MTA and EMD
Yildirim et al. [56]	Human 127 carious primary molars (32 w/EMD)	Pulpotomy	ZOE cement + GIC + SSC	3, 6, 12, 18 and 24 months	90.6% clinical and 78.1% radiographic success	No significant difference in success rates between formocresol, MTA, PC

*Note:* EMD, emdogain, enamel matrix derivative.

Abbreviations: CaOH, calcium hydroxide; GIC, glass ionomer cement; MTA, mineral trioxide aggregate; PC, portland cement; PRP, platelet‐rich plasma; SSC, stainless steel crown; ZOE, zinc oxide eugenol.

Despite these promising findings, several practical limitations constrain the clinical application of current EMD formulations in endodontics. The gel‐based delivery system may compromise the integrity of the coronal seal, increasing the risk of microleakage and reducing the stability of the overlying restoration [54]. In addition, the viscous and flowable nature of the material can hinder controlled placement and condensation of restorative materials, potentially resulting in inconsistent application [55]. Histological observations further suggest that pulpal tissue may proliferate into voids created by the gel, thereby interfering with the formation of a continuous hard tissue bridge [39, 40]. Moreover, diffusion of the material into adjacent healthy pulp tissue may induce unintended calcification, raising concerns regarding the preservation of normal pulp architecture and function—an essential determinant of long‐term treatment success [46].

From a practical perspective, EMD is supplied in a syringe with a limited working time of approximately 2 h following opening, which may affect its cost‐effectiveness and clinical convenience [54, 55].

Finally, the generalisability of the existing evidence base remains limited. Many experimental studies do not adequately replicate clinical conditions, particularly with respect to the pre‐operative inflammatory status of the pulp. In several cases, teeth included in studies exhibited minimal or no pulpal inflammation, which does not reflect the typical clinical presentation of teeth requiring DPC [39, 40, 42, 53]. Under such conditions, more conservative approaches, such as indirect pulp capping or selective caries removal, may have been more appropriate [59]. Furthermore, discrepancies between histological and clinical outcomes—especially over the long term—highlight the need for more robust, longitudinal clinical trials. Future research should therefore focus on standardising experimental protocols, improving delivery systems and conducting well‐designed long‐term clinical studies to better define the role of EMD in vital pulp therapy. Enhancements in formulation and handling characteristics will be critical to increasing its clinical acceptance and optimising its therapeutic potential in endodontic applications.

### 2.6. EMD and Recession Defects

Numerous clinical trials have assessed the management of buccal Cairo Type I (Miller Class I‐II) periodontal recession. Many used manual probe measurements, which may have caused inaccuracies. Clinical calibration and proper sample size calculations could have reduced these limitations [60–66]. Together, these studies show that the adjunctive use of EMD enhances KT formation and leads to improved, more stable root coverage (RC) outcomes [60–66]. Roman et al. [67] evaluated the effectiveness of sub‐epithelial connective tissue grafts (SCTGs) and coronally advanced flaps (CAFs), both with and without adjunctive EMD. After 1 year, RC percentages were comparable, ranging from 82% to 89%, for both treatments. A study by Dias et al. [68] investigated EMD’s effect on periodontal healing after RC surgery. It included two groups: the test group received CAF and SCTG with EMD, and the control group received CAF with SCTG alone. The test group showed significantly greater average RC at 86%, compared to 66% in the control group (*p* = 0.008). Additionally, EMD with CAF‐SCTG significantly increased vascular endothelial growth factor (VEGF) expression [68]. These results suggest EMD may promote angiogenesis and enhance healing after mucogingival surgery.

Although EMD is promising for managing periodontal recession, most studies have short follow‐up periods and small sample sizes. Mercado et al. [69] conducted a comprehensive trial with 80 patients over 3 years. The SCTG‐EMD group achieved complete RC in 66.4% of cases, compared to 50% in the SCTG‐only group. Patients treated with SCTG‐EMD also had higher rates of root and recession coverage, greater KT width and reported less pain 2 weeks after surgery. The study concluded that adding EMD improves long‐term outcomes for multiple adjacent recessions in both upper and lower anterior teeth. However, longer‐term studies of at least 3 years are needed to fully assess the benefits of EMD compared to SCTG alone [69].

Predictable RC in Cairo Type III (Miller Class III–IV) recessions is challenging due to factors such as loss of interdental bone and soft tissue, increased avascular surfaces, root prominence and a reduced periosteal bed [70, 71]. Nart et al. [72] treated 14 Miller Class II and III (Cairo Type I‐II) recessions in 10 patients using a SCTG with a CAF. Complete RC was achieved in five (71.4%) Miller Class II sites and three (42.9%) Class III defects (69). These results suggest that SCTG combined with CAF is effective for RC in mandibular incisors with Class II and III recession defects, even without the additional benefits of EMD [72].

Henriquez et al. [73] conducted a split‐mouth study focusing on Miller Class III recessions (Cairo Type II) involving 12 patients. The results conveyed that the SCTG‐EMD group demonstrated superior mean RC (MRC) of 70% compared to 54.8% for the SCTG‐alone group after a 12‐month follow‐up [73]. Conversely, Aroca et al. [70] were unable to demonstrate the adjunctive effect of EMD in their study, which comprised 20 patients undergoing a modified tunnel/SCTG procedure with or without EMD at multiple Miller Class III (Cairo Type II) recession sites. Although both groups exhibited significant improvement in recession coverage, there were no notable differences in outcomes, with MRC reported at 82% in the EMD group and 83% in the control group.

Regarding Miller Class IV recessions, classified as Cairo Type III, most evidence comes from case reports and series [74]. Vergara and Caffesse [75] used an “envelope” graft technique on 12 Miller Class IV (Cairo Type III) defects in patients over 40 years old. They achieved a MRC of 62.7% and a complete RC rate of 16% after 6 months. Other studies have reported partial RC and CAL gain [76–79]. Although some evidence suggests that EMD may benefit the management of Class III–IV recession defects, more clinical studies with larger samples and longer follow‐ups are needed.

A single randomised clinical trial has investigated the management of Type III Cairo (Class III–IV Miller) periodontal recessions specifically affecting the lower anterior teeth [78]. This study enrolled 41 patients and followed them for 3 years, evaluating 156 teeth divided into two groups: a test group treated with SCTG combined with EMD (79 teeth) and a control group treated with SCTG alone (77 teeth). At the 36‐month follow‐up, the test group demonstrated significantly greater improvement in recession coverage (from 5.71 ± 0.58 mm to 1.57 ± 0.85 mm) compared to the control group (from 5.94 ± 0.46 mm to 2.51 ± 0.62 mm). The width of KT also increased more substantially in the test group (from 1.51 ± 0.26 mm to 4.18 ± 0.34 mm) than in the control group (from 1.65 ± 0.21 mm to 2.90 ± 0.20 mm). Moreover, patients in the test group experienced significantly less postoperative pain at 2, 7 and 14 days after surgery. These findings suggest that the addition of EMD is beneficial for the management of advanced recession (Type III Cairo/Class III–IV Miller), as it is associated with greater KT gain and more stable long‐term outcomes.

Reaching consensus regarding the benefits of using EMD as an adjunct to the established gold standard for RC, which is the bilaminar technique of SCTG with a CAF, remains challenging. However, EMD has been shown to promote improved healing, as measured by visual analogue scales (VASs) and increases in KT. Clinicians frequently employ EMD as an adjunct in complex cases that require enhanced wound healing, since this biologically active material appears to enhance the regenerative capacity of local tissues.

### 2.7. Histological Assessment of Human Biopsies After Recession Management With EMD

Histological evidence from human studies on the treatment of recession defects, both with and without EMD, has demonstrated variable success in achieving full or partial periodontal regeneration [2, 67, 73, 79–81]. McGuire et al. [2] investigated nine teeth scheduled for extraction as part of orthodontic treatment. Of these, seven teeth received a combination of EMD–SCTG–CAF. This intervention resulted in observable periodontal regeneration, as evidenced by the formation of cementum, new bone and periodontal attachment. Despite limitations in the current literature, there is consensus that EMD promotes significant regeneration when used in conjunction with SCTG [2]. This combination improves both support and stability in the management of recession defects [73, 81]. Table 2 presents current histological evidence regarding regenerative outcomes in the management of recession defects using EMD. Among 35 teeth extracted following mucogingival surgery with EMD application, 57% (20 teeth) exhibited complete periodontal regeneration, as indicated by the presence of PDL and cementum attached to the newly formed alveolar bone. In contrast, 28.5% (10 teeth) developed a long junctional epithelium upon histological examination, a result consistent with outcomes from mucogingival surgeries performed without biologically active materials. The 57% success rate in achieving true periodontal regeneration with EMD underscores the need for further research. Additional histological studies are warranted to determine whether EMD or other biologically active materials, when combined with various mucogingival surgical techniques, enhance regeneration following recession coverage.

**Table 2:** Published human histological results following EMD used in periodontal recession management.

Study	Regeneration (number of teeth)	Connective tissue adhesion/including bone formation (# of teeth)	Long junctional epithelium (# of teeth)	Ankylosis/resorption (# of teeth)
Heijl [79]	1	—	—	—
Mellonig [82]	1	—	—	—
Sculean et al. [83]	3	4	—	—
Sculean et al. [84]	2	—	—	—
Yukna and Mellonig [85]	3	3	4	—
Carnio et al [86]	1	3	—	—
McGuire et al [2]	8	—	—	1
Total # and %	19/56%	10/29%	4/12%	1/3%

### 2.8. EMD in Periodontal Defects

Beyond its established adjunctive role in RC procedures, EMD has been extensively utilised in the management of a range of periodontal defects, including intrabony and furcation defects. EMD has been investigated as a standalone regenerative modality, in comparison with guided tissue regeneration (GTR) using barrier membranes, and as an adjunct to surgical access therapy [87–91]. Regenerative approaches are generally recommended for intrabony defects exceeding 3 mm in depth, typically employing either barrier membranes or EMD delivered via papilla‐preserving techniques with minimal flap elevation to optimise wound stability and healing [87].

### 2.9. EMD in Intrabony Defects

A variety of surgical approaches have been developed for the management of intrabony defects, including the modified Widman flap (MWF), papilla preservation techniques (PPTs), minimally invasive surgical technique (MIST) and modified MIST (M‐MIST). Comparative evidence indicates that extended PPT and MIST yield similar clinical outcomes when both are combined with adjunctive EMD [88]. Contemporary surgical strategies emphasise minimisation of tissue trauma, enhancement of wound stability and preservation of the regenerative space to optimise both clinical outcomes and patient comfort [89–91].

A substantial body of evidence supports the adjunctive benefit of EMD in intrabony defect therapy. Multiple studies have demonstrated greater PD reduction and CAL gain when EMD is combined with surgical debridement compared with MWF or PPT alone [79, 92–99]. However, these findings are not entirely consistent. Studies utilising MIST have reported significant clinical and radiographic improvements with the surgical approach alone, without additional benefit from EMD [91, 100].

More recently, flapless approaches have been proposed as minimally invasive alternatives to MIST. These techniques utilise atraumatic gingival retraction and magnification to facilitate root surface debridement while reducing surgical time [101, 102]. Early evidence suggests that flapless procedures combined with EMD can achieve outcomes comparable to MIST; however, greater bone fill has been reported in MIST‐only groups [101]. Both approaches demonstrate continued bone fill between 12 and 24 months, highlighting the importance of long‐term evaluation. In contrast, Aimetti et al. [102] reported significantly greater CAL gain with flapless surgery combined with EMD (3.9 ± 1.1 mm) compared with flapless surgery alone (3.0 ± 1.2 mm).

Defect morphology remains a critical determinant of regenerative success. EMD demonstrates reduced efficacy in one‐ and two‐wall defects, where CAL gains are generally lower than those in three‐wall defects [96, 103]. This limitation is largely attributed to the gel‐like consistency of EMD, which lacks the structural capacity to maintain space for regeneration. Furthermore, EMD has been associated with less bone regeneration compared with GTR in certain contexts [83, 104]. Consequently, combination approaches incorporating grafting materials have been developed to enhance space maintenance and prevent the collapse of the mucoperiosteal flap.

### 2.10. EMD Combined With Bone Graft Materials in Intrabony Defects

Current European Federation of Periodontology (EFP) guidelines recommend the use of grafting materials in regenerative therapy for intrabony defects [87]. However, the additional benefit of EMD when combined with grafts remains a subject of ongoing debate [105]. Evidence indicates that combining EMD with biphasic calcium phosphate (BCP) significantly improves PD reduction and CAL gain in one‐ and two‐wall defects, with outcomes maintained for up to 3 years [106–108]. Similarly, the use of EMD with xenogeneic grafts, including Bio‐Oss, Cerabone and demineralised porcine bone matrix (DPBM), has been shown to enhance PD reduction, CAL gain and defect fill across a range of defect morphologies, with some studies demonstrating stability of results for up to 10 years [91, 107, 109, 110]. In addition, the combination of EMD with autogenous bone grafts has resulted in significantly greater improvements in PD reduction, CAL gain and probing bone levels compared with EMD alone [111].

### 2.11. EMD and GTR in Intrabony Defects

Clinical evidence suggests that EMD achieves outcomes comparable to GTR in terms of PD reduction and CAL gain, particularly in two‐ and three‐wall intrabony defects [84, 93, 103, 112, 113]. A recent meta‐analysis by Miron et al. [114] reported slightly greater PD reduction (0.51 mm) and CAL gain (0.19 mm) with GTR compared with EMD; however, these differences were not statistically significant. Despite comparable soft tissue outcomes, several studies have demonstrated greater bone regeneration with GTR [83, 104].

The choice between EMD and GTR is influenced by clinical considerations and associated risk profiles. Membrane exposure remains a common complication of GTR and may lead to bacterial contamination and compromised aesthetic outcomes, including papilla loss [92, 112]. Consequently, EMD is often preferred in aesthetically sensitive regions where the preservation of soft tissue architecture is critical. Conversely, GTR may offer advantages in non‐aesthetic areas and in deeper, non‐contained defects with fewer remaining bony walls, such as one‐ and two‐wall defects [103].

### 2.12. EMD in Furcation Defects

The application of EMD in furcation defect management has been widely investigated, particularly in mandibular Class II defects. Overall, the literature demonstrates that EMD improves clinical parameters; however, its superiority over conventional or alternative regenerative approaches remains variable.

Chitsazi et al. [115] reported that open flap debridement (OFD) with adjunctive EMD resulted in significantly greater horizontal CAL gain and improved horizontal and vertical defect resolution compared with OFD alone at 6 months, although PD reduction and vertical attachment gain were similar between groups. Similarly, Casarin et al. [116, 117] found that while OFD + EDTA and OFD + EDTA + EMD produced comparable improvements in most clinical parameters, the addition of EMD increased the likelihood of furcation conversion from Class II to Class I and complete closure. Combining regenerative approaches may further enhance clinical outcomes. Aimetti et al. [118] demonstrated improved horizontal PD reduction and radiographic bone fill when EMD was combined with autogenous bone grafts compared with OFD, although complete closure was not achieved. Jaiswal and Deo [119] reported that a combined approach using EMD, demineralised freeze‐dried bone allograft (DFDBA), and a bioresorbable membrane resulted in significantly greater PD reduction and attachment gain than DFDBA with membrane alone or OFD.

However, not all studies have demonstrated a clear advantage of EMD. Peres et al. [120] and Queiroz et al. [121] reported similar clinical and radiographic improvements between EMD, graft materials and combination therapies, with no statistically significant differences, although higher rates of defect improvement were often observed in EMD‐containing protocols. Limiroli et al. [122] reported favourable clinical and radiographic outcomes with EMD combined with deproteinised bovine bone mineral (DBBM), with fewer complications compared to membrane‐based therapy, although differences were not statistically significant.

In more advanced furcation defects, Donos et al. [123] demonstrated that EMD, GTR and their combination produced comparable improvements in Class III mandibular furcation defects, with no clear superiority among modalities. Similarly, Jepsen et al. [124] reported that EMD achieved significantly greater horizontal defect reduction and higher rates of complete closure compared with bioabsorbable membranes, along with reduced postoperative morbidity. Meyle et al. [125] observed comparable clinical improvements between EMD and membrane therapy, with less gingival recession and absence of measurable bone resorption in EMD‐treated sites. Hoffmann et al. [126] further highlighted the influence of patient‐related factors, reporting greater improvements with EMD in non‐smokers and older patients, while no significant differences were observed in smokers or younger individuals. Table 3 provides a summary of published studies investigating the application of EMD for the treatment of mandibular furcation defects.

**Table 3:** Summary of included studies on EMD for mandibular furcation involvement.

Authors	Year	Title	Journal	Study type
Limiroli et al. [122]	2023	Regenerative surgery of mandibular class II furcation defects: A comparison of two techniques in a randomised clinical trial with 3D CBCT measurements at 24 months	International Journal of Periodontics & Restorative Dentistry	RCT
Queiroz et al. [127]	2017	Furcation therapy with enamel matrix derivative: Effects on the subgingival microbiome	Journal of Periodontology	RCT
Queiroz et al. [121]	2016	Enamel matrix protein derivative and/or synthetic bone substitute for treatment of mandibular class II buccal furcation defects	Clinical Oral Investigations	RCT
Jaiswal and Deo [119]	2013	Evaluation of the effectiveness of enamel matrix derivative, bone grafts, and membrane in treatment of mandibular class II furcation defects	International Journal of Periodontics & Restorative Dentistry	RCT
Peres et al. [120]	2013	Hydroxyapatite/β‐TCP and enamel matrix derivative for proximal class II furcation defects	Journal of Clinical Periodontology	RCT
Casarin et al. [117]	2010	Enamel matrix derivative proteins for proximal class II furcation involvements	Journal of Clinical Periodontology	RCT
Casarin et al. [116]	2008	Double‐blind RCT of enamel matrix derivative proteins for proximal class II furcation involvements	Journal of Clinical Periodontology	RCT
Chitsazi et al. [115]	2007	Open flap debridement with and without enamel matrix derivative in mandibular degree II furcation	Clinical Oral Investigations	RCT
Hoffmann et al. [126]	2006	Multicentre trial comparing enamel matrix derivative and membrane treatment of buccal Class II furcation involvement (Part III)	Journal of Clinical Periodontology	Multicentre RCT
Aimetti et al. [118]	2005	Treatment of mandibular class II furcation defects using amelogenins and autologous bone	Minerva Stomatologica	Case Reports
Meyle et al. [125]	2004	RCT comparing enamel matrix derivative and membrane treatment of buccal Class II furcation involvement (Part II)	Journal of Periodontology	Multicentre RCT
Jepsen et al. [124]	2004	RCT comparing enamel matrix derivative and membrane treatment of buccal class II furcation involvement (Part I)	Journal of Periodontology	Multicentre RCT
Donos et al. [123]	2004	Clinical evaluation of enamel matrix derivative and bioresorbable membrane in degree III mandibular furcation involvement	International Journal of Periodontics & Restorative Dentistry	Case series

The microbiological effects of EMD therapy have also been explored in furcation defects. Queiroz et al. [127] demonstrated that EMD, alone or in combination with grafting, promoted a shift toward a healthier subgingival microbiome, with sustained reductions in key periodontal pathogens such as *Filifactor alocis*, *Parvimonas micra* and *Selenomonas noxia* over a 6‐month period, compared with grafting alone. These findings suggest enhanced microbial stability despite comparable clinical outcomes across treatment modalities.

Regenerative therapy for mandibular furcation defects has consistently demonstrated clinically significant improvements, irrespective of the treatment modality employed. Within this framework, the adjunctive application of EMD is frequently associated with enhanced horizontal clinical attachment gain, improved defect resolution and, in certain cases, conversion of furcation involvement to a less severe classification when compared with OFD alone. However, these advantages are not uniformly observed across all clinical parameters as differences in PD reduction and vertical attachment gain are often not statistically significant. The potential benefit of combining EMD with bone grafts or barrier membranes has been widely investigated, particularly for non‐contained defects where maintenance of regenerative space is critical. Nevertheless, the additional clinical advantage of EMD in such combination approaches remains inconsistent. While some studies report numerically improved outcomes or reduced complication rates when EMD is used adjunctively, these findings are not consistently reproduced, suggesting that the effectiveness of combination therapy may depend on the defect characteristics and surgical factors.

Comparisons between EMD and GTR indicate broadly similar efficacy in terms of key clinical outcomes. Despite this overall equivalence, EMD may offer advantages in specific contexts, including greater horizontal defect reduction, reduced postoperative morbidity and improved outcomes in selected patient subgroups. These attributes may be particularly relevant in clinical situations where minimising surgical complications and preserving soft tissue architecture are priorities.

From a microbiological perspective, EMD therapy appears to facilitate a shift toward a more stable, health‐associated subgingival microbiota. This effect may contribute to the long‐term stability of regenerative outcomes by promoting a favourable ecological environment for periodontal healing.

Collectively, the available evidence supports the use of EMD as an effective regenerative modality in the management of both intrabony and furcation defects. Although EMD is associated with significant improvements in clinical parameters, its therapeutic efficacy is influenced by multiple factors, including defect morphology, surgical technique and the adjunctive use of grafting materials. In many clinical scenarios, EMD achieves outcomes comparable to GTR while demonstrating a more favourable complication profile, particularly in aesthetically sensitive regions where preservation of soft tissue architecture is essential. Furthermore, combination approaches incorporating graft materials may enhance regenerative outcomes in non‐contained defects by improving space maintenance and stabilising the regenerative environment.

### 2.13. EMD in Peri‐Implant Disease Management

EMD has been explored in the management of peri‐implant diseases, including peri‐implant mucositis and peri‐implantitis, due to its regenerative and anti‐inflammatory properties. In peri‐implant mucositis, adjunctive application of EMD following mechanical debridement has demonstrated favourable short‐term outcomes. Clinical studies report approximately a 50% reduction in bleeding on probing (BOP) and a PD reduction of 1.5–3 mm at 3 months post‐treatment [128]. These clinical improvements are accompanied by significant reductions in pro‐inflammatory cytokines, including interleukin (IL)‐6 and IL‐17, suggesting that EMD exerts an immunomodulatory effect in peri‐implant soft tissues [128].

In contrast, peri‐implantitis represents a more complex condition that often requires surgical intervention. While non‐surgical approaches such as mechanical debridement, photodynamic therapy and systemic or local antibiotic administration may occasionally resolve inflammation, they are generally insufficient for advanced lesions, which frequently necessitate surgical access and, in some cases, regenerative therapy [129–133]. A critical component of peri‐implantitis management is the decontamination of the implant surface following flap elevation and the removal of infected granulation tissue. Numerous decontamination methods have been proposed, including saline, lasers, hydrogen peroxide, citric acid, EDTA, tetracycline and chlorhexidine; however, current evidence does not support the superiority of any single approach [134–137].

Clinical findings in the use of EMD in peri‐implantitis management remain heterogeneous (Table 4). Surgical protocols incorporating bone grafts in combination with EMD have demonstrated promising outcomes in terms of bone regeneration, whereas non‐surgical applications of EMD tend to yield improvements primarily in short‐term clinical parameters. The variability in treatment protocols, along with the limited number of long‐term RCTs, restricts definitive conclusions regarding their efficacy. Two principal clinical protocols have been described for the use of biologically active materials such as EMD in peri‐implantitis management: application of the material alone and combination with a carrier bone graft [130, 131, 139, 141]. In the single‐material approach, an RCT evaluated debridement and decontamination of 26 implants using gauze and saline, followed by the application of EMD [139]. After 12 months, the EMD‐treated group demonstrated a higher prevalence of Gram‐positive aerobic bacteria and increased marginal bone levels. Although PD and BOP did not differ significantly between EMD and control groups at 1 year, the EMD group exhibited a higher implant survival rate at 5 years (85% versus 75%) [131].

**Table 4:** Clinical studies on EMD and peri‐implantitis.

Author and year	Study design	Clinical result	Limitation
Froum et al. (2012, 2015) [130, 138]	Clinical (cohort)	1.77 mm bone gain, 5.1 mm PD reduction over 2–10 years (EMD + grafts/PDGF)	No control group; retrospective
Kashefimehr et al. (2016) [128]	RCT	EMD reduced BOP by 50% and PD by 1.5 mm at 3 months (non‐surgical)	Short follow‐up; unblinded
Isehed et al. (2016, 2018) [131, 139]	RCT	No significant PD/BOP differences vs. control; EMD improved 5‐year survival (85%)	High risk of bias; unclear randomisation
Esberg et al. (2019) [140]	Clinical (RCT)	EMD reduced inflammatory PICF proteins; linked to implant survival	Small cohort (*n* = 25)
Mercado et al. (2018) [141]	Prospective cohort	PD reduction (8.9 mm → 3.5 mm), bone gain (6.92 mm → 2.60 mm) over 3 years	No control group; combined therapy (EMD + DBBM)

In the combined‐material protocol, biologically active agents such as EMD or platelet‐derived growth factor (PDGF) are used in conjunction with bone graft materials. This approach has been associated with sustained clinical improvements and enhanced regenerative outcomes [130, 141]. Long‐term follow‐up studies ranging from 2 to 10 years report a mean PD reduction of 5.1 mm and an average bone gain of 1.77 mm when EMD or PDGF is combined with grafts in moderate to severe peri‐implantitis cases [130, 138]. Similarly, Mercado et al. [141] demonstrated that the use of EMD with DBBMC and doxycycline resulted in stable reductions in PD (from 8.9 to 3.5 mm) and improvements in bone levels (from 6.92 to 2.60 mm) over a 3‐year period.

Despite these encouraging findings, recent RCTs have questioned the additional benefit of EMD in regenerative peri‐implantitis therapy. Regidor et al. [142] reported no significant advantage of EMD when used in combination with bone grafts and resorbable membranes compared with surgical intervention alone. Furthermore, Moldovan et al. [143] concluded that while EMD may improve short‐term clinical outcomes, its superiority over other regenerative materials, such as bone grafts, remains unproven.

Overall, the current body of evidence suggests that EMD may contribute to improved peri‐implantitis treatment outcomes through its potential to enhance osteogenesis, modulate inflammation and support soft tissue healing. However, its clinical effectiveness appears to be influenced by treatment protocols and defect characteristics. Further high‐quality, multicenter randomised clinical trials with long‐term follow‐up are required to establish the definitive role of EMD in the management of peri‐implant diseases.

### 2.14. EMD in Alveolar Ridge Preservation (ARP)

Following tooth extraction, the alveolar socket undergoes substantial dimensional changes, characterised by both vertical and horizontal ridge resorption, ultimately resulting in a significant reduction in ridge volume. These alterations can compromise subsequent implant placement and prosthetic outcomes [92–95]. To mitigate these changes, a wide range of soft and hard tissue preservation strategies have been developed. Soft tissue management approaches include the use of SCTG, free gingival grafts, soft tissue substitutes and resorbable membranes aimed at promoting socket closure and preserving the soft tissue profile [144–147]. These procedures are frequently performed using a flapless approach, particularly in cases where the buccal plate remains intact. A key objective of such interventions is to achieve complete socket closure while facilitating the formation of KT.

In parallel, various grafting materials have been employed to support hard tissue regeneration within the extraction socket, including autogenous bone, allografts, xenografts and alloplastic substitutes, each demonstrating variable clinical outcomes [147–150]. More recently, biologically active agents—such as bone morphogenetic proteins, PDGFs and EMD, have been incorporated into ridge preservation protocols to enhance regenerative potential when used in conjunction with bone substitutes [147, 151]. Clinical evidence evaluating the efficacy of EMD in ARP remains limited and somewhat heterogeneous. Mercado et al. [147] compared the use of EMD combined with DBBMC to DBBMC alone in maxillary anterior ridge preservation. Radiographic outcomes demonstrated no significant differences between groups in terms of ridge width and height preservation. However, histomorphometric analysis revealed a markedly higher proportion of new bone formation in the EMD group (45.1%) compared with the control group (16.5%), alongside a reduction in the residual graft material (20.3% versus 36.8%) [147]. These findings suggest that EMD may enhance bone quality and graft turnover despite its limited effects on overall ridge dimensions. Similarly, Lee et al. [151] evaluated EMD in combination with DBBM‐C compared with DBBM‐C alone in posterior maxillary ridge preservation. Cone‐beam computed tomography (CBCT) assessments at 5 months postoperatively demonstrated no significant differences in horizontal or vertical bone loss between the two groups. Notably, the EMD‐treated group experienced a shorter duration of postoperative discomfort, with reductions in both pain (by 1.2 days) and swelling (by 1.06 days) relative to the controls [151]. These findings highlight a potential role for EMD in improving patient‐centred outcomes, particularly in the early healing phase.

Alkan et al. [152] further investigated the effects of EMD compared with Bio‐Oss Collagen in ridge preservation by assessing peri‐implant crevicular fluid prostaglandin E_2_ (PGE_2_) levels and implant stability quotient (ISQ) values. The EMD group demonstrated significantly elevated PGE_2_ levels at 1 month, indicative of an early inflammatory response that subsided by 3 months. Concurrently, significant increases in ISQ values were observed from baseline to 3 months, suggesting enhanced implant stability and osseointegration. These findings support the hypothesis that EMD may positively influence early bone healing dynamics and implant integration.

Overall, the current body of evidence suggests that EMD may enhance bone quality by promoting osteogenesis and facilitating graft integration. However, its effectiveness in preventing post‐extraction ridge resorption appears limited. The anti‐inflammatory properties of EMD may contribute to accelerated resolution of postoperative symptoms, although this effect is not consistently reported across all studies (Table 5).

**Table 5:** Clinical studies on EMD and alveolar ridge preservation.

Author (year)	Study design	Sample size	Intervention	Control	Follow‐up	Key findings	Limitations
Mercado et al. (2021) [147]	RCT	42 patients (21/group)	DBBMC + EMD	DBBMC alone	4 months	• 45.1% new bone vs. 16.5% (*p* < 0.00001) • No significant radiographic differences	• Short follow‐up
Lee et al. (2020) [151]	RCT	28 patients	DBBM‐C + collagen membrane + EMD	DBBM‐C + collagen membrane	5 months	• Reduced pain duration by 1.2 days (*p* = 0.008) • No difference in ridge dimensions	• Small sample size • Subjective pain reporting
Alkan et al. (2016) [152]	RCT	12 patients (24 sockets)	EMD	Bio‐Oss collagen	3 months	• Higher initial PGE_2_ levels (*p* = 0.008) • Improved ISQ values (*p* = 0.012)	• Very small sample • No histology
Atieh et al. (2023) [153]	RCT	18 patients (25 sites)	DBBM + EMD	DBBM alone	6 months	• 54.5% sites with <1 mm height loss vs. 14.3% (*p* = 0.03) • More postoperative discomfort	• Uneven molar/premolar distribution No histology
Lee et al. (2020) [151]	RCT	30 patients	DBBM‐C + EMD	DBBM‐C alone	5 months	• Short duration • No ridge dimension benefit	• Anterior sites only • No control group benefit

Interpretation of the available data is complicated by substantial heterogeneity in study design, including variations in the EMD concentration, carrier materials, surgical protocols and outcome measures. Additionally, most clinical studies are characterised by small sample sizes (typically 12–30 participants) and short follow‐up periods (generally 3–6 months), limiting the generalisability of findings. The absence of robust long‐term data on implant survival and success rates further restricts definitive conclusions.

In summary, while EMD demonstrates potential in enhancing bone quality and soft tissue healing in ARP, its impact on maintaining ridge dimensions remains inconclusive. Future research should focus on well‐designed, adequately powered RCTs with long‐term follow‐up to establish standardised treatment protocols and to evaluate the clinical and cost‐effectiveness of EMD in routine practice.

### 2.15. EMD on Wound Healing

Wound healing is a complex and highly regulated biological process involving cellular attachment, migration, proliferation and differentiation, ultimately leading to new tissue formation and wound closure [154, 155]. EMD has been shown to significantly influence multiple phases of this process through its interactions with host cells and the modulation of key molecular pathways. At the cellular level, EMD enhances the production of transforming growth factor‐beta (TGF‐ß) when interacting with gingival fibroblasts and PDL cells, thereby promoting cellular proliferation and matrix formation [156–159]. In addition, EMD plays a critical role in angiogenesis, an essential component of effective wound healing. This effect is mediated through activation of the extracellular signal‐regulated kinase (ERK) pathway and the stimulation of capillary‐like structure formation from human umbilical vein endothelial cell (HUVEC) spheroids in a dose‐dependent manner [160]. Furthermore, EMD upregulates VEGF, thereby enhancing neovascularisation at the wound site [160–163].

EMD also contributes to hard tissue healing by modulating bone metabolism. It increases osteoprotegerin (OPG) gene expression and protein synthesis while downregulating receptor activator of nuclear factor kappa‐B ligand (RANKL) expression, favouring osteoblast differentiation and inhibiting osteoclastogenesis [164–166]. In osteoblast‐like cell lines (MG‐63), EMD has been shown to activate the phosphoinositide 3‐kinase (PI3K) signalling pathway, which is associated with enhanced osteoblast migration and regenerative activity during wound healing [167].

The regenerative effects of EMD on both soft and hard tissues have been substantiated across molecular, animal and human clinical studies [168–173]. In a rat model, EMD significantly improved oral mucosal wound healing by promoting angiogenesis and collagen fibre formation. These effects were associated with increased expression of TGF‐ß1, VEGF, matrix metalloproteinase‐1 (MMP‐1) and fibronectin, all of which are critical mediators of tissue repair [168]. In vitro studies using human PDL cells have further identified a low molecular weight fraction of EMPs, referred to as ‘Fraction C’, which upregulates endothelial markers and enhances angiogenic activity, suggesting potential applications in advanced regenerative therapies [171].

Clinical evidence also supports the beneficial role of EMD in wound healing. In a double‐masked, split‐mouth randomised clinical trial involving 28 patients, the combination of EMD with polyglycolic acid as a carrier resulted in significantly improved soft tissue healing compared with polyglycolic acid alone as assessed by VAS scores at 3 weeks postoperatively [169].

The recognition that EMD exerts favourable effects on oral epithelial and connective tissue healing has prompted further investigation into its potential applications beyond the oral cavity, particularly in cutaneous wound healing. The skin, as the largest organ of the human body, serves as a critical protective barrier against environmental insults, including microorganisms and radiation, while also playing a key role in thermoregulation. Structurally, it comprises a highly organised system of epithelial, connective, vascular and immune components, sharing many cellular and functional characteristics with oral tissues. Cells such as epithelial cells, fibroblasts, endothelial cells and inflammatory cells participate in both active and passive aspects of tissue repair, analogous to the processes observed in the oral environment.

Given these similarities, EMD has been investigated in in vitro, animal, and human studies for its capacity to enhance wound healing in skin tissues. Its demonstrated ability to modulate inflammation, promote angiogenesis and stimulate ECM formation supports its potential as a biologically active agent in both oral and extraoral wound healing applications.

### 2.16. EMD and Dermal Applications

Recent translational research has highlighted the potential of EMD to enhance wound healing beyond the oral environment, as demonstrated in both in vitro and animal studies. These findings have supported the exploration of EMD for extra‐oral applications, largely due to its ability to form a temporary ECM that facilitates tissue repair. In this context, a commercially available formulation, Xelma (Mölnlycke Health Care, Gothenburg, Sweden), has been developed specifically for non‐oral use. Xelma functions as an ECM protein‐based therapy, providing a provisional scaffold that promotes cell adhesion and stimulates granulation tissue formation when applied to chronic, non‐healing wounds, including venous leg ulcers, diabetic ulcers and rheumatoid ulcers [174]. This approach represents a biologically‐driven strategy to improve wound healing outcomes in difficult‐to‐manage clinical scenarios.

### 2.17. Venous Leg and Diabetic Ulcers

Venous leg ulcers and diabetic ulcers are chronic wounds primarily associated with impaired venous return, resulting in increased venous pressure and subsequent tissue breakdown [129]. The development of these ulcers is strongly linked to risk factors such as chronic venous insufficiency, obesity, deep vein thrombosis, advanced age, immobility and prolonged standing, which collectively account for approximately 70%–90% of venous leg ulcer cases [175].

Clinical studies have demonstrated that EMD may provide therapeutic benefits in the management of these chronic wounds. In a study involving 117 patients, treatment with EMD in combination with compression therapy resulted in a 33.8% reduction in wound size, compared with a 25.6% reduction observed with placebo plus compression. Notably, in ulcers larger than 10 cm^2^, EMD treatment achieved a 25% reduction in wound size, compared to only 7.9% in the placebo group, indicating a more pronounced benefit in larger, more complex wounds [176]. Hard‐to‐heal ulcers, defined as those exceeding 10 cm^2^ in size and persisting for more than 6 months, appear to derive particular benefit from EMD therapy [176].

Similarly, another clinical study including 83 participants reported a mean ulcer size reduction of 33.1% in the EMD‐treated group, compared with 11.1% in patients managed with compression bandaging alone (*p* = 0.03). In addition to greater reductions in wound size, the EMD group demonstrated a higher proportion of clinical improvement and a lower incidence of moderate‐to‐high exudate levels. Furthermore, patients receiving EMD reported greater reductions in pain, both at rest and during dressing changes, compared with those of controls [176].

Beyond these findings, EMD has also been shown to stimulate healing in wounds that are unresponsive to conventional therapies and to contribute to improvements in patient‐reported quality of life [177]. Collectively, these results support the potential role of EMD as an adjunctive treatment in the management of chronic dermal wounds, particularly in cases where standard therapeutic approaches have proven to be insufficient.

### 2.18. EMD and Malignant Cells

Due to its growth factor–like effects on fibroblasts [178, 179], osteoblasts [147, 157] and ECM cells [180], EMD has also been investigated for its potential influence on malignant cell behaviour, although the available evidence remains inconsistent [5, 6, 181–186]. Collectively, experimental data suggest that EMD may exert divergent and sometimes opposing effects depending on the cell type and experimental context.

In vitro and in vivo studies using murine models have demonstrated that EMD can upregulate MMPs (MMP‐2 and MMP‐9) in tongue carcinoma cell lines. This increase in MMP expression was associated with enhanced cellular migration and a potential facilitation of metastatic spread in mice bearing human tongue carcinoma xenografts [5]. These findings raise concerns that EMD, through its ECM–modulating activity, may contribute to tumour progression in certain settings. Consequently, it has been suggested that EMD should be used with caution, or potentially avoided, in patients with oral carcinoma or premalignant lesions, given its capacity to influence pathways implicated in tumour invasion [5].

The biological plausibility of these observations is supported by the established role of MMPs in tumorigenesis. Elevated MMP activity contributes to multiple stages of cancer progression, including angiogenesis, activation of latent growth factors, and degradation of ECM components, thereby facilitating tumour invasion and metastasis [187, 188]. Clinically, increased MMP expression is associated with more aggressive tumour phenotypes and poorer prognostic outcomes [187]. In this context, EMD has been shown to stimulate malignant cell activity by upregulating MMP‐1 [167] as well as MMP‐2 and MMP‐9 [5], further supporting a potential pro‐invasive effect under specific conditions.

In addition, TGFβ1 is a central cytokine in carcinogenesis [181], contributing to epithelial‐to‐mesenchymal transition (EMT), which represents an early and critical step in malignant transformation and tumour progression [182]. EMD has been reported to stimulate TGFβ1 production, thereby potentially enhancing MMP‐mediated invasion through TGF‐β–dependent signalling pathways [183]. This mechanistic link further supports the hypothesis that EMD may, in certain contexts, facilitate tumour‐associated processes through coordinated modulation of TGF‐β and MMP activity [183].

However, not all evidence supports a pro‐tumorigenic role for EMD. Takayama et al. [6] investigated its effects on malignant cells using the MCF‐7 breast cancer cell line and reported that EMD functions as an anti‐adhesive agent, significantly reducing cancer cell attachment to bone. This finding is of potential relevance given the predilection of breast carcinoma for skeletal metastasis, particularly to the rib cage. Nevertheless, the clinical implications of this observation remain uncertain due to the limitations inherent in in vitro experimental models.

Similarly, Kawase et al. [189] examined the effects of EMD on oral epithelial cells using SCC25 cells derived from human tongue squamous cell carcinoma. Their findings indicated that EMD exerts anti‐proliferative effects on epithelial cells, acting in a cytostatic rather than cytotoxic manner. This effect may be beneficial in periodontal regeneration by limiting epithelial downgrowth and thereby favouring connective tissue cell repopulation.

Taken together, these studies highlight a complex and context‐dependent biological profile of EMD in relation to malignant and premalignant cells. While some evidence suggests potential pro‐invasive effects mediated through MMP and TGFβ1 pathways, other findings indicate anti‐adhesive and anti‐proliferative properties in specific epithelial and cancer cell lines. These apparently opposing effects have led to uncertainty regarding their application in oncological contexts. Accordingly, further well‐designed preclinical studies and prospective clinical trials are required to clarify the safety profile of EMD and to determine its potential influence on malignant transformation, tumour progression and metastatic behaviour.

## 3. Conclusion

Three decades after its commercial introduction, EMD remains a cornerstone biologically active agent in periodontal and regenerative therapy. Its biomimetic and host‐modulatory properties have enabled a broad spectrum of clinical applications, ranging from adjunctive use in mucogingival procedures and RC to periodontal regeneration, peri‐implant defect management, ARP and the enhancement of wound healing in both oral and extra‐oral tissues. The accumulated evidence supports its capacity to promote favourable biological responses, including modulation of inflammation, stimulation of angiogenesis and enhancement of both soft and hard tissue regeneration.

Despite these well‐established benefits, the field of regenerative dentistry continues to evolve with the emergence of novel biologically active materials. Recently introduced chairside agents, such as Regenfast (polynucleotides and hyaluronic acid), Gem21 (recombinant human PDGF and rhPDGF) and Regroth (basic fibroblast growth factor), offer alternative mechanisms to enhance tissue healing and regeneration. These materials may broaden the therapeutic armamentarium available to clinicians and potentially address limitations associated with current regenerative approaches, particularly in the management of critical‐size defects and complex soft tissue deficiencies.

Nevertheless, while these newer agents show promising early outcomes, robust long‐term clinical evidence remains limited. In this context, EMD, particularly in its widely used formulation, continues to represent a reliable and extensively validated option, supported by decades of clinical research and longitudinal data. Although its position as the preferred adjunctive biologically active material may face increasing competition, it is likely to remain a mainstay in periodontal and regenerative practice until the long‐term efficacy and comparative effectiveness of emerging biologics are clearly established.

## Author Contributions

Faustino Mercado led conceptualisation, project administration, data curation and draughted both the original and revised manuscripts, and had full access to all of the data in this study, taking complete responsibility for the integrity of the data and the accuracy of the data analysis. Carolina Loch and Ahmad Mustafa contributed to data curation and research, focusing on the biology of EMD in periodontal defects and implant therapies and assisted with draughting and revising the manuscript. Euna Lee and Michelle Hecker contributed to data curation and research on the endodontic effects of EMD and assisted with writing and revising the manuscript.

## Funding

No financial support, benefits or funding from commercial or official entities were received in connexion with this article. Open access publishing facilitated by University of Otago, as part of the Wiley ‐ University of Otago agreement via the Council of Australasian University Librarians.

## Disclosure

All authors have read and approved the final version of the manuscript.

## Conflicts of Interest

The authors declare no conflicts of interest.

## Data Availability

The data supporting the findings of this study are available within the article, as detailed in the relevant tables presented in the manuscript.
